# Assessing the causal impact of leisure-time physical activity and screen time on lifespan: a Mendelian randomization study

**DOI:** 10.1186/s11556-026-00406-0

**Published:** 2026-03-06

**Authors:** Zhu Liduzi Jiesisibieke, C. Mary Schooling

**Affiliations:** 1 School of Public Health, Li Ka Shing Faculty of Medicine, The University of Hong Kong, Hong Kong, China; 2 Graduate School of Public Health and Health Policy, The City University of New York, New York 10027 New York, USA

**Keywords:** Physical activity, Screen time, Lifespan, Mendelian randomization

## Abstract

**Objectives:**

Observational studies have consistently shown physical activity associated with lower mortality. Randomized controlled trials to confirmAuthors’ contributions the value of physical activity for lifespan in the general population are challenging to conduct. To address this gap, we conducted a Mendelian Randomization (MR) study, using the largest available suitable studies and control outcomes.

**Method:**

We conducted a two-sample MR study using summary statistics in European populations. We used single nucleotide polymorphisms (SNPs) strongly (*p* < 5 × 10^− 8^), and independently (r^2^ < 0.001) predicting leisure-time moderate to vigorous intensity physical activity (*N* = 608,595) and SNPs similarly predicting inactivity (*N* = 526,725, leisure-time screen time) to obtain inverse variance weighted estimates. Lifespan was proxied by parental attained age (current age or age at death). We adjusted for education using multivariable MR. Waist circumference and whole-body fat mass were control outcomes. Sensitivity analyses included the weighted median, MR-Egger, MR-PRESSO and MRlap.

**Results:**

Leisure-time moderate to vigorous intensity physical activity was associated with longer lifespan for men (1.41 years per doubling the odds of being physically active in leisure time, 95% confidence interval (CI) 0.21 to 2.62) and women (1.68 years, 95% CI 0.12 to 3.25). Conversely, leisure-time screen time was associated with shorter lifespan, which was less evident after adjusting for education. As expected, leisure-time moderate to vigorous intensity physical activity reduced and screen time increased waist circumference and whole-body fat mass, which remained after adjusting for education.

**Conclusions:**

Leisure-time moderate to vigorous intensity physical activity may extend lifespan, while the role of leisure-time screen time is less clear. Questions remain about the optimal type, duration, intensity, and frequency of physical activity.

**Supplementary Information:**

The online version contains supplementary material available at 10.1186/s11556-026-00406-0.

## Highlights

- Physical activity contributes to longer lifespan.
- Benefits of physical activity are similar for men and women.
- Efforts should be made to ensure equitable access to physical activity opportunities, considering social and cultural norms.

**Supplementary Information:**

The online version contains supplementary material available at 10.1186/s11556-026-00406-0.

## Introduction

Physical inactivity is one of the four target behaviours identified by the World Health Organization (WHO) which should be addressed to improve population health [1]. The WHO has established guidelines for physical activity (both aerobic and anaerobic) [2], emphasizing that promoting physical activity is crucial for achieving several of the 2030 Sustainable Development Goals [3]. The WHO’s 2020 guidelines recommend all adults engage in 150–300 min of moderate-intensity, or 75–150 min of vigorous-intensity physical activity per week [2]. Physical activity triggers multi-organ responses, involving the skeletal system, heart and lungs [4–6].

Epidemiological evidence linking physical activity to health can be traced back to the 1950s. A seminal study compared bus conductors with bus drivers, as bus conductors had more occupational physical activity than bus drivers. The study found a lower risk of coronary heart disease in the bus conductors [7, 8]. Subsequently, many observational studies have confirmed that physical activity is associated with lower mortality [9–11]. However, observational studies are open to confounding, because physical activity requires time and energy as well as resources and access to facilities, so is likely influenced by socioeconomic position and other unmeasured or unmeasurable confounders. Trials that have comprehensively assessed the effect of physical activity on mortality in the general population are lacking [12]. One randomized controlled trial conducted in 1,567 older participants (mean age: 72.8) found no evidence that high-intensity interval training or moderate-intensity continuous training affected all-cause mortality [13], but likely was underpowered. A systematic review and meta-analysis concerning 165,000 former athletes found that, overall, athletes lived longer, but male anaerobic athletes did not. Evidence about women and anaerobic athletes [14] is lacking. No experimental or quasi-experimental study has assessed the role of physical activity in lifespan for men and women from the general population.

Mendelian randomization (MR), i.e., instrumental variable analysis with genetic instruments, is an alternative epidemiological approach for drawing causal inferences [15]. MR studies use genetic information (i.e., single nucleotide polymorphisms (SNPs)) as instruments to predict exposures, which largely obviates confounding. A previous MR study found no association of moderate to vigorous intensity physical activity with extreme longevity, likely due to limited power [16]. To address this gap, we conducted an MR study to assess the associations of leisure-time moderate to vigorous intensity physical activity and screen time, as an indicator of sedentary behaviour [17], with lifespan, based on parental attained age given that lifespan is heritable [18], as in previous studies [19]. Parental attained age has more variability and hence more power than participant lifespan. Additionally, given the average age at recruitment to the UK Biobank was 57 years, to extend the age range we included age at recruitment as an outcome, as previously [20–22]. Age at recruitment is a measure of survival to late middle-age because the genetics of harmful exposures inevitably become rarer with increasing age at recruitment. Given men and women differ in patterns of physical activity and lifespan, we considered them together and separately. Finally, we used well-established consequences of physical activity and inactivity as positive control outcomes, i.e., waist circumference [23, 24] and whole-body fat mass [24, 25].

## Methods

### Study design

**Fig. 1:**
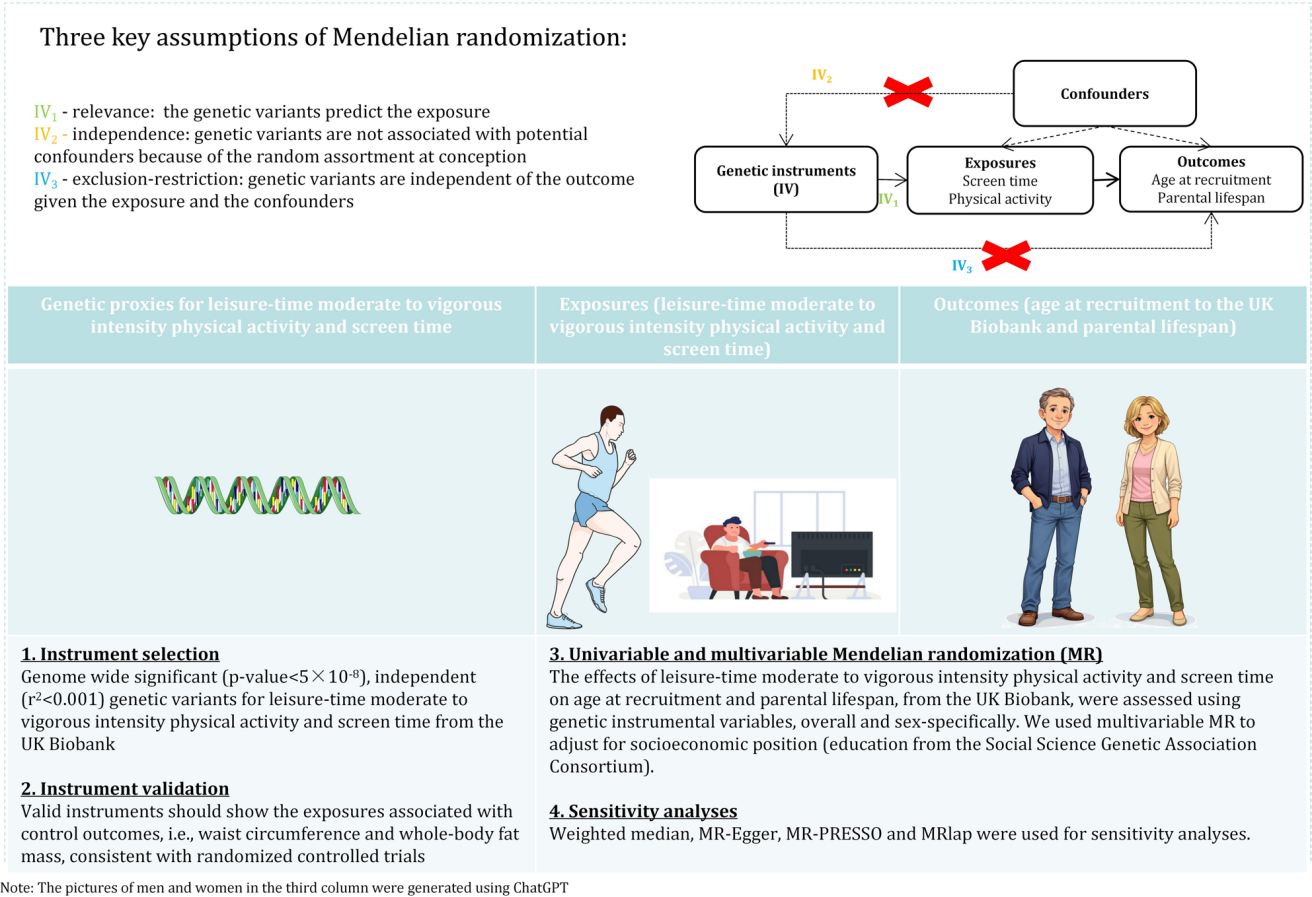
Study design diagram

Instrumental variable analysis has stringent assumptions (relevance, independence and exclusion restriction) [26, 27]. Use of strong genetic instruments addresses relevance. Use of genetic instruments also addresses independence, because genetic endowment is less open to confounding than directly observed exposures [27]. The genetic studies used were also designed to obviate confounding by population structure (independence). We used sensitivity analysis to assess horizontal pleiotropy (exclusion restriction). Although, MR is much less open to confounding than many other observational studies, we also adjusted for socio-economic position (SEP), because physical activity is likely affected by SEP. Figure 1 illustrates the study design.

### Genetic instruments for leisure time moderate to vigorous intensity physical activity and screen time

Genetic instruments for leisure-time moderate-to-vigorous intensity physical activity in two groups were obtained from studies with varying definitions; including in the largest study, i.e. the UK Biobank, $\:\ge\:$20 minutes per week versus less than 20 minutes or inactive, were taken from a meta-analysis of studies in people of European ancestry [17]. We obtained genetic instruments for leisure-time television watching, originally called leisure-time screen time because the underlying studies largely predate mobile phones or widespread use of home computers [17]. We used strong (p-value < 5 × 10⁻⁸), independent (r²<0.001, distance < 10,000 kb) genetic instruments for leisure-time moderate-to-vigorous intensity physical activity (*n* = 608,595; ~44% men; ~48% active, with similar proportions in men (~ 49%) and women (~ 47%) [17], obtained from GWAS summary statistics of Europeans adjusted for age, age², and principal components (Table 1). Leisure-time screen time (*n* = 526,725) was also derived from this study.

**Table 1:** Data sources used in this MR study

Trait	Data source	Ancestry	Sex	Sample size	Adjusted covariates	*R* ^2^	F statistics in univariable MR
Leisure-time moderate-to-vigorous intensity physical activity	Wang, 2022, Nature Genetics	European ancestry	Men and women	608,595	Age, age^2^, principal components	0.45%	37.2
Leisure-time screen time	Wang, 2022, Nature Genetics	European ancestry	Men and women	526,725	Age, age^2^, principal components	3.12%	39.3
Education	Lee, 2018, Nature Genetics, UK Biobank	European ancestry	Men and women	766,345	Genetic principal components		
Smoking initiation	Liu, 2019, GWAS & Sequencing Consortium of Alcohol and Nicotine use	European ancestry	Men and women	607,291	Genetic principal components		
Alcohol intake frequency	Ben Elsworth, UK Biobank	White British	Men and women	462,346	Sex and genotyping array		
Overall health rating	UK Biobank (Neale lab)	White British	Men and women	361,194	Age, age^2^, sex, age×sex, age^2^ ×sex, and first 20 principal components		
Waist circumference	Elsworth, 2018, UK Biobank	European	Men and women	462,166	Genotype array, sex and the first 10 principal components		
Whole body fat mass	Elsworth, 2018, UK Biobank	European	Men and women	454,137	Genotype array, sex and the first 10 principal components		
Age at recruitment	UK Biobank (Neale lab)	White British	Men	167,020	First 20 principal components		
Age at recruitment	UK Biobank (Neale lab)	White British	Women	194,174	First 20 principal components		
Father’s attained age	UK Biobank, Pilling et al., 2017	European	Men*	415,311	Age, indicators of assessment center, array type		
Mother’s attained age	UK Biobank, Pilling et al., 2017	European	Women*	412,937	Age, indicators of assessment center, array type		
Parental attained age	UK Biobank and LifeGen, Timmers et al., 2019	European	Men and women	1,012,240	Genotyping batch and array, the first 40 principal components of relatedness, sex, and study-specific covariates		

*Sex refers to the outcome in the parents not the sex of the participants

### Genome wide associations studies for control outcomes

The UK Biobank was used to obtain genetic associations with the control outcomes, i.e., waist circumference (*n* = 462,166) and whole-body fat mass (*n* = 454,137) adjusted for genotype array, sex, and principal components (first 10) (Table 1).

### Genome wide association studies for the outcome, i.e., measures of lifespan 

Genetic associations with sex-specific parental attained age were from the UK Biobank, a population-based study in Great Britain (recruited 2006 to 2010), mean age ~ 57 years, with slightly more women than men [28, 29]. Genotyping used the UK BiLEVE and UKB Axiom arrays [30] as explained in detail elsewhere [29, 31]. Quality-controlled overall and sex-specific genetic associations with attained age of parents, fathers (age at death: 317,652, current age: 97,659) and mothers (age at death: 246,941, current age: 165,996)) were taken from a study of the UK Biobank (Table 1) [32] adjusted for age, indicators of assessment centre and array type. Lifespan is heritable [18]. Exclusion criteria included death of mother before 57 years or of father before 46 years [32] and adoption [32]. For ease of interpretation, log protection ratios were converted into years of participant life using a validated approximation, i.e., multiply by -10 and by 2.5863 for mothers and 2.2869 for fathers [33]. Offspring only inherit half of each parent’s genetic endowment [33], so the effect size in the offspring only represents half of the true effect, hence this conversion. Quality-controlled genetic associations with age at recruitment (an indicator of survival to late middle-age) to the UK Biobank were taken from Neale Lab UK Biobank (http://www.nealelab.is/uk-biobank) (Table 1). Finally, we also used sex-combined lifespan from Timmers et al. [33] (*n* = 1,012,240) based on the UK Biobank and LifeGen, adjusted for genotyping batch and array, the first 40 principal components of relatedness, sex, and study-specific covariates.

### Potential confounders

Although MR should be robust to confounding we also considered measures of socioeconomic position and health status as potential confounders. We used education (*n* = 766,345) [34] and health status (*n* = 361,194) from the UK Biobank (http://www.nealelab.is/uk-biobank) (Supplementary Fig. 1). We also assessed whether smoking initiation (*n* = 607,291), from the GWAS & Sequencing Consortium of Alcohol and Nicotine use [35], and alcohol drinking frequency (*n* = 462,346) from the UK Biobank were confounders (Supplementary Fig. 1).

### Statistical analysis

Genetic instruments were aligned on the same effect allele for exposure and outcome, additionally using allele frequency and strand alignment for palindromic SNPs.

### Univariable Mendelian randomization

Inverse variance weighting (IVW) estimates were used as the main analysis, with the weighted median (WM) and MR-Egger estimates used as sensitivity analyses. The WM is valid when > 50% of the weight is from valid genetic variants [36]. MR-Egger allows for directional pleiotropy, but still needs to satisfy the Instrumental Strength Independent of Direct Effect (InSIDE) assumption [26], i.e. any pleiotropic effects of the instruments are independent of their associations with the exposure. Despite use of large samples, bias towards the confounded estimate from sample overlap is possible for MR-Egger estimates, when I^2^_GX_ is low [37]. MRlap was used to assess bias from overlapping samples. MR-PRESSO was used to identify outliers. We converted the log odds of being physically active into a doubling in the odds of being physically active by multiplying by 0.693 (natural logarithm of two).

### Multivariable Mendelian randomization

Multivariable MR (MVMR) uses genetic variants associated with multiple potentially related exposures to assess the direct effects of multiple potentially related exposures [38, 39] or to assess potential mediators in two-step MR [40]. In this study, we used MVMR to adjust for SEP (proxied by education) to mitigate potential bias due to assortative mating [41, 42]. To comprehensively understand the role of physical activity given lifestyle traits are often related, we examined whether the genetic instruments for physical activity also showed associations with other common lifestyle factors. Lifestyle factors that showed evidence of influencing physical activity were included in the MVMR to mitigate potential horizontal pleiotropy. As sensitivity analysis we also used the multivariable weighted median (MV-WM) [43] and MR-Egger (MV-MR-Egger) [44]. Significance of differences by sex were obtained from a z-test [45].

### Instrument strength, validity assessment and power calculation

Instrument strength was assessed from F-statistics, estimated as (β²/var(β)) [46] for univariable MR and using the Sanderson-Windmeijer method for multivariable MR [47], with F-statistics > 10 indicating adequate instrument strength [48]. We estimated R², the proportion of exposure variance explained by the genetic instruments, as: R² = 2 * EAF * (1 – EAF) * β², where β is the standardized genetic association with the exposure and EAF is effect allele frequency [49]. Heterogeneity between SNPs was assessed using Cochran’s Q [47]. Power was estimated using the approximation that the MR sample size needed is that required to detect an association for exposure on outcome divided by r^2^ for genetic instruments on exposure [50, 51].

### Software and ethical considerations

We used the R packages “TwoSampleMR” (version: 0.6.1), “MendelianRandomization” (version: 0.10.0), “MRlap” (version 0.0.3) and “MRPRESSO” (version 1.0) to facilitate the MR analysis, “metafor” (version: 4.6-0) to assess differences by sex and “forestplotter” (version: 1.1.2) in R (4.4.1, 2024-06-14 ucrt) to create graphics. We only used publicly available data in this study, so no ethics approval was needed.

## Results

### Instrument strength and validity

After aligning the genetic variants across studies, we obtained 16 independent genome-wide significant SNPs for leisure-time moderate to vigorous intensity physical activity and 113 for leisure-time screen time. The minimum F statistic was above 10 (Table 1). The variance of for the causal effects for the causal effects leisure-time moderate to vigorous intensity physical activity and leisure-time screen time explained by the instruments was 0.45% and 3.12% respectively. The detectable difference for the IVW estimate in parental lifespan and age at recruitment, based on 80% power and a 5% alpha level, is presented in Supplementary Table 1.

### Assessment of assumptions

**Fig. 2:**
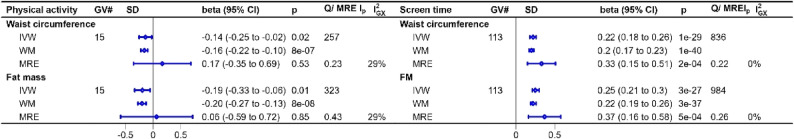
Mendelian randomization estimates for the causal effects of leisure-time moderate to vigorous intensity physical activity and screen time on waist circumference and whole-body fat mass. Note: Causal effects of a doubling in the odds of being physically active (proxied by genetically predicted leisure-time moderate-to-vigorous intensity physical activity ; n= 608,595) and of leisure-time screen time (per hour increase; n=526,725) from Wang et al. (2022) (including some participants from the UK Biobank) on the control outcomes of waist circumference (n=462,166) and whole-body fat mass (n=454,137) from the UK Biobank. *Abbreviations*: *GV#* Genetic variants number, *SD* Standard deviation, *MRE Ip* p-value for MR-Egger intercept

**Fig. 3:**
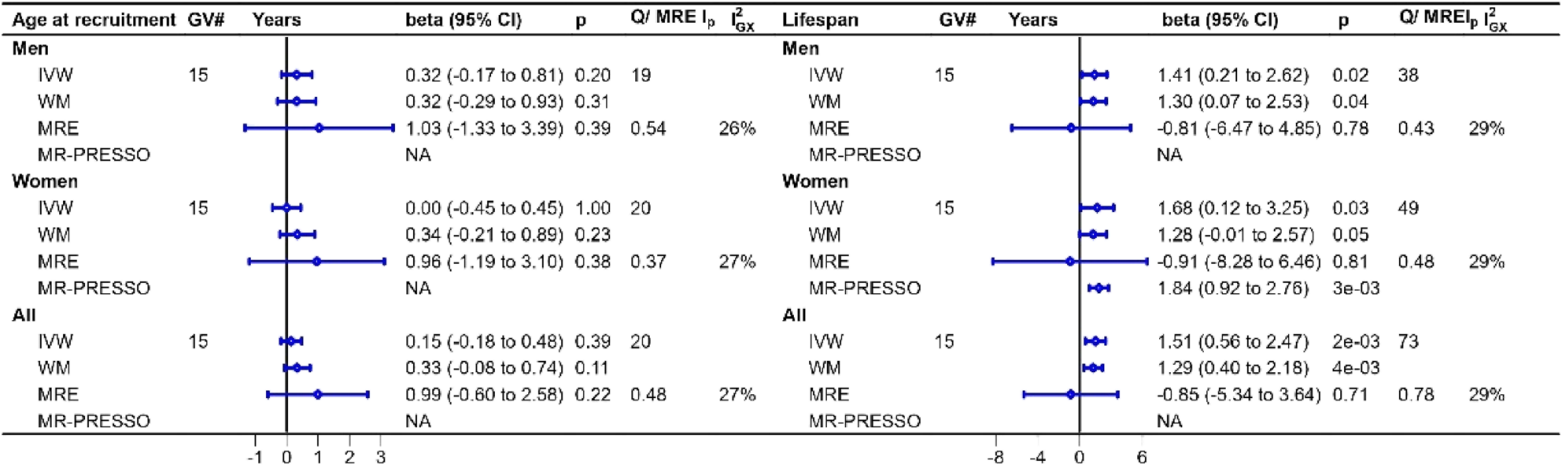
Mendelian randomization estimates for the causal effects of leisure-time moderate to vigorous intensity physical activity on age at recruitment and lifespan. Note: Causal effects of a doubling in the odds of being physically active (proxied by genetically predicted leisure-time moderate-to-vigorous intensity physical activity; n= 608,595) from Wang et al. (2022) (including some participants from the UK Biobank) on age at recruitment in men (n=167,020), women (n=194,174) and overall (n=361,194) and parental attained age (fathers=415,311), (mothers=412,937) and overall (n=828,248) from the UK Biobank. *Abbreviations*: *GV#* Genetic variants number, *SD* Standard deviation, *MRE Ip* p-value for MR-Egger intercept

**Fig. 4:**
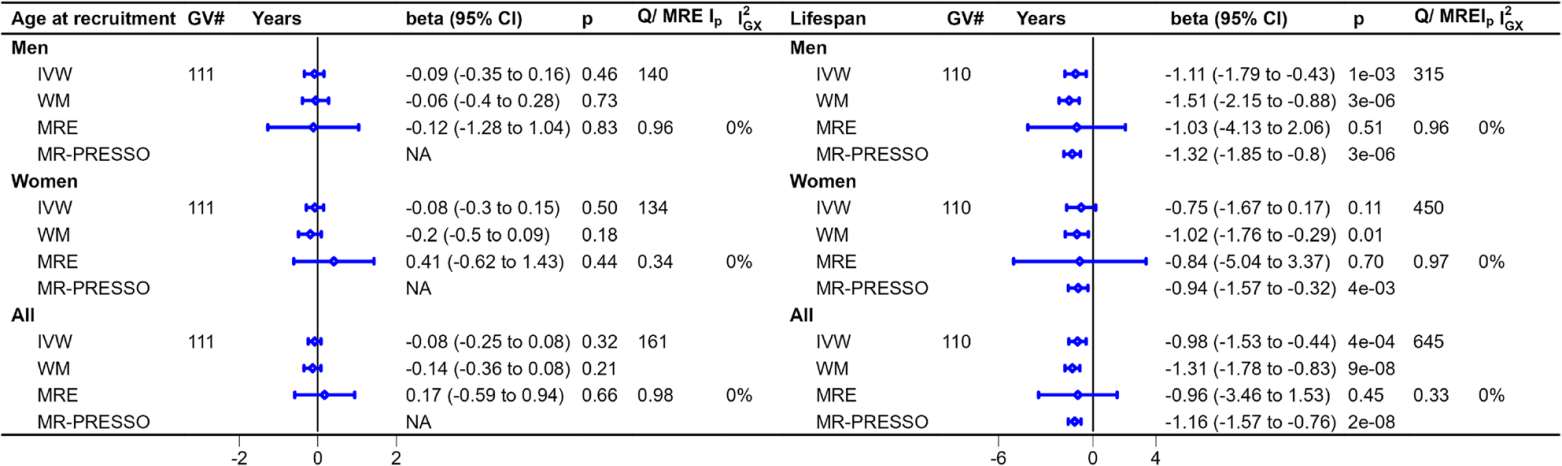
Mendelian randomization estimates for the causal effects of leisure-time screen -time on age at recruitment and lifespan. Note: Causal effects of leisure-time screen time (per hour increase; n=526,725) from Wang et al. (2022) (including some participants from the UK Biobank) on age at recruitment in men (n=167,020), women (n=194,174) and overall (n=361,194) and parental attained age for men (n=415,311), women (n=412,937) and overall (n=828,248) from the UK Biobank. *Abbreviations*: *GV#* Genetic variants number, *SD* Standard deviation, *MRE Ip* p-value for MR-Egger intercept

The F-statistics met the criteria thereby addressing relevance. We adjusted for education to address potential horizontal pleiotropy. We did not adjust for health status, because the F-statistic in multivariable analysis was too low and the associations between health status and physical activity may be bidirectional (Supplementary Fig. 1). The p-value for the MR-Egger intercept did not show violations of the exclusion restriction assumption (Figs. 2, 3 and 4).

### Associations of leisure-time moderate to vigorous intensity physical activity and screen time with the control outcomes

As expected, leisure-time moderate-to-vigorous intensity physical activity was associated with lower waist circumference and lower fat mass (Fig. 2). Sensitivity analyses, using WM and MR-Egger gave consistent estimates. After adjusting for education, leisure-time moderate-to-vigorous intensity physical activity remained associated with waist circumference and lower fat mass; leisure-time screen time remained positively associated with higher waist circumference and whole-body fat mass (Supplementary Fig. 2).

### Univariable MR estimates for leisure-time moderate to vigorous intensity physical activity on measures of lifespan

Leisure-time moderate to vigorous intensity physical activity was not clearly associated with survival to UK Biobank recruitment (Fig. 3). MRlap did not indicate major differences of interpretation (Supplementary Table 2). Leisure-time moderate to vigorous intensity physical activity was associated with longer lifespan for men (1.41 years, 95% CI: 0.21 to 2.62) and women (1.68 years, 95% CI: 0.12 to 3.25) using IVW estimates with no sex difference (p-value = 0.79) (Fig. 3). MR-PRESSO estimates were similar for women and no outliers were identified for men. MRlap estimates indicated some possible underestimations especially for women (Supplementary Table 2). MR-Egger estimates differed from the IVW estimates; MR-Egger is sensitive to random measurement error and weak instrument bias, given the I^2^_GX_ was low, indicating lack of the instrument strength for MR-Egger. So, we used multivariable MR [52]. Replication using a larger of GWAS of lifespan in both sexes gave a similar interpretation (Supplementary Fig. 3).

### Univariable MR estimates for associations of leisure-time screen time with measure of lifespan

Leisure-time screen time was not associated with survival to UK Biobank recruitment (Fig. 4). MRlap suggested little differences in interpretation (Supplementary Table 2). Leisure-time screen time was associated with shorter lifespan for men (-1.11 years, -1.79 to -0.43) but not women (-0.75 years, -1.67 to 0.17) using IVW estimates with no sex difference (*p* = 0.54) (Fig. 4). Estimates from MR-PRESSO were similar (Fig. 4), MRlap estimates suggested underestimation (Supplementary Table 2).

### Multivariable MR estimates adjusted for education

After adjusting for education, the estimates were more consistent across methods. Leisure-time moderate to vigorous intensity physical activity was associated with longer lifespan for men (1.26 years, 95% CI: 0.38 to 2.14), women (1.22 years, 95% CI: 0.34 to 2.10) and overall (1.24 years, 95% CI: 0.62 to 1.86) (Supplementary Fig. 4), with no sex difference (p-value = 0.89). Leisure-time screen time was no longer clearly associated with lifespan (Supplementary Fig. 4), with no sex difference (p-value: 0.38).

## Discussion

Consistent with previous observational evidence showing physical activity associated with lower mortality [9, 10], using a design less open to confounding, we add by showing leisure-time moderate to vigorous intensity physical activity associated with longer lifespan for men and women, after adjustment for education. As expected, leisure-time moderate to vigorous intensity physical activity was also associated with lower waist circumference and whole-body fat mass, while the opposite association was observed for leisure-time screen time, which remained evident after adjustment for education.

### Comparison with previous studies

Previous MR studies of physical activity on cardiovascular diseases have been inconsistent [53, 54]. Accelerometer-measured physical activity reduced breast and colorectal cancer risk [55], and moderate to vigorous intensity physical activity reduced type 2 diabetes risk [56]. A previous MR study found directionally consistent associations of moderate to vigorous intensity physical activity (*n* = 377,324) with a measure of survival, i.e., living beyond the 90th survival percentile (*n* = 11,262, odds ratio: 1.89, 95% CI 0.53 to 6.70) but was underpowered [16]. Traditional observational studies have generally suggested physical activity reduces mortality [57–60]. However, physical activity requires time and effort as well as being easier for those in good health, so these studies are open to confounding. A study comparing within middle-aged and older twin pairs to obviate confounding found no effect of physical activity on cardiovascular disease risk or mortality risk [61]. Studies in older people may also be open to selection bias, which may bias towards the null or even the reverse. Here, we assessed physical activity on lifespan at younger ages, proxied by survival to recruitment, and at older ages, proxied by parental lifespan, and consistently found benefits (Fig. 3).

Previous cohort studies have found a harmful association of self-reported sedentary behaviors (such as watching television, driving, and using computers) with all-cause mortality [62]. Here, leisure-time screen time (i.e., television watching), a form of sedentary behavior, was associated with a shorter lifespan for men and overall. However, this association disappeared after adjusting for education. Extensive television watching is often linked to poorer financial and health conditions [63], which suggests socioeconomic factors may confound this association [64, 65].

The association of physical activity with lifespan is complex and multifaceted. Physical activity reduces body fat, which may partly explain its beneficial effects on health and longevity [66–68]. However, anaerobic exercise increases basal metabolic rate [69], which may increase risk of cancer [70]. Conversely, physical activity activates AMP-activated protein kinase (AMPK) [71], which generally promotes health and longevity [72, 73]. However, it is unclear whether moderate-intensity physical activity is sufficient to trigger AMPK activation consistently [74], especially in trained athletes with high metabolic thresholds [75–77]. Benefits may arise from transitioning from a sedentary state to one of movement, regardless of intensity [78, 79]. More nuanced measures of physical activity may be needed to capture all its benefits.

### Strengths

This study has several strengths. First, the large sample size of the most recently available and largest GWAS provide unparallel statistical power to investigate the effect of leisure-time moderate to vigorous intensity physical activity and screen time on lifespan using an MR design. Second, using age at recruitment and parental lifespan instead of participant mortality reduces selection bias and has more statistical power than participant lifespan. Third, we used control outcomes to assess the validity of the instruments for leisure-time moderate to vigorous intensity physical activity and screen time. Fourth, the MR design allows investigation of lifelong effects of leisure-time moderate to vigorous intensity physical activity and screen time on lifespan in the general population instead of relatively acute effects in specific groups. Fifth, we used MR-PRESSO and MRlap to identify outliers and assess effects of overlapping samples respectively, which gave similar interpretations. Sixth, we considered confounding by socioeconomic position by adjusting for education. We did not adjust for self-reported health because it is unclear whether self-rated health is a cause or a consequence of physical activity (Supplementary Fig. 1) and the F-statistic in the multivariable MR was too low. Finally, estimates are given in years of life which may be more easily interpretable than relative risk.

### Limitations

There are several limitations in this study. First, participants may have overreported their leisure-time moderate to vigorous intensity physical activity and underreported leisure-time screen time. However, consistent over- and under-reporting affects magnitude and power, more than direction. Second, not all potential participants survived to recruitment, and early parental deaths were excluded, hence use of UK Biobank age at recruitment as an outcome. Third, the UK Biobank and other studies used here recruited volunteers. However, associations found in the UK Biobank are similar to those obtained from population representative studies [80, 81]. Fourth, some non-paternity is possible but is unlikely to be related to leisure-time moderate to vigorous intensity physical activity or screen time, so any bias is likely towards the null. Fifth, overlapping samples were used, but MRlap indicated little resulting bias. Sixth, biological mechanisms influencing physical activity and inactivity are not completely understood so we could not use a potentially more reliable biologically driven strategy for genetic instrument selection [82]. Seventh, the definition of “active” varied in the original GWAS and did not necessarily meet the WHO guidelines, which may dilute the estimated effects compared with recommended activity levels. Eighth, physical activity in the original GWAS is a binary exposure, which may lose some variability of the exposure. Ninth, adjusting for all potential confounders (such as health status, smoking and alcohol use) as well as education would make the genetic instruments too weak, so results should be interpreted with caution. Tenth, we did not provide triangulation of evidence because many purely observational studies using the UK Biobank have shown physical activity associated with reduced all-cause mortality [83–87] and evidence from randomized controlled trials or quasi-experimental studies is limited. Consistency by age in these previous observational studies suggests possible bias [87]. Eleventh, use of European populations limits generalizability although the underlying biological mechanisms should be similar. Twelfth, changes between generations in the UK, such as less smoking and heart disease nowadays may mean the role of these factors in years of life is overestimated. Finally, we could not assess effects of vigorous physical activity because a sufficiently large GWAS is not available. However, leisure-time moderate to vigorous intensity physical activity may be a more practical target for public health initiatives.

### Policy implications

Our study suggests that physical activity has benefits for lifespan. To promote physical activity for all, interventions should target improvements in walkability and access to facilities given physical activity is influenced by socio-economics and environmental factors and people are sensitive to their surroundings [88]. In addition, consideration should be given to ensuring equitable access to physical activity, while considering social and cultural norms [89] and feasibility. Financial constraints and tiredness remain the primary barriers to undertaking physical activity [90].

## Conclusion

Our findings provide some support for the WHO guidelines by demonstrating that leisure-time moderate to vigorous intensity physical activity is associated with longer lifespan for men and women. Conversely, leisure-time screen time possibly showed an opposite effect. Nevertheless, questions remain about the optimal type, duration, intensity, and frequency of physical activity to optimize health.

## Supplementary Information

Supplementary Material 1.

## Data Availability

This study used data from the MR-base plat form (https://www.mrbase.org/), UK Biobank (http://www.nealelab.is/uk-biobank/), OpenGWAS (https://opengwas.io/), the Neale lab (https://www.nealelab.is/uk-biobank) and GWAS Catalog (https://www.ebi.ac.uk/gwas/).
