# Supplementary material for: Assessing the causal impact of leisure-time physical activity and screen time on lifespan: a Mendelian randomization study
Source: Eur Rev Aging Phys Act. 2026 Mar 6;23:14. doi: 10.1186/s11556-026-00406-0 (PMC13088598; doi:10.1186/s11556-026-00406-0)
Supplement: Supplementary file 1 — Supplementary Material 1. [file 11556_2026_406_MOESM1_ESM.docx]

**Supplementary Figure 1. Assessing potential confounders on leisure-time moderate to vigorous intensity physical activity (left) and leisure-time moderate to vigorous intensity physical activity on potential confounders (right)**

**
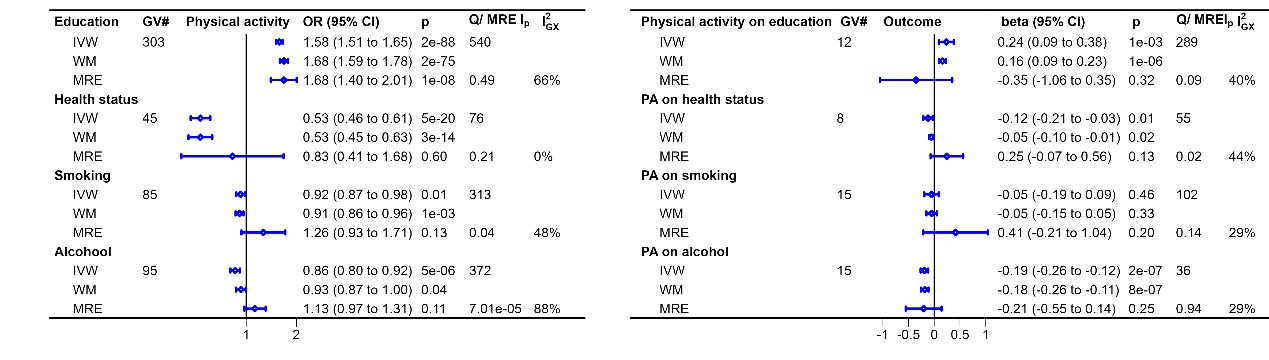
**

**Note:**

**Causal effects of education (n= 766,345) from the Social Science Genetic Association Consortium, poorer health status (self-reported overall health rating; n= 361,194) from Neale lab, smoking initiation (n=607,291) from the GWAS & Sequencing Consortium of Alcohol and Nicotine use, alcohol intake frequency from the UK Biobank on being physically active (proxied by genetically predicted leisure-time moderate to vigorous intensity physical activity; n= 608,595) from Wang et al. (2022) (including some participants from the UK Biobank) (left) and the causal effects of a doubling in the odds of being physically active (proxied by genetically predicted leisure-time moderate to vigorous intensity physical activity; n= 608,595) on education, poorer health status, smoking initiation and alcohol intake frequency (right).**

**Abbreviations: GV#: genetic variants number; SD: standard deviation; MRE Ip: p-value for MR-Egger intercept**

**Supplementary Figure 2.** **Multivariable Mendelian randomization estimates for the causal effects of leisure-time moderate to vigorous intensity physical activity and screen time adjusted for education on waist circumference and whole-body fat mass**

*
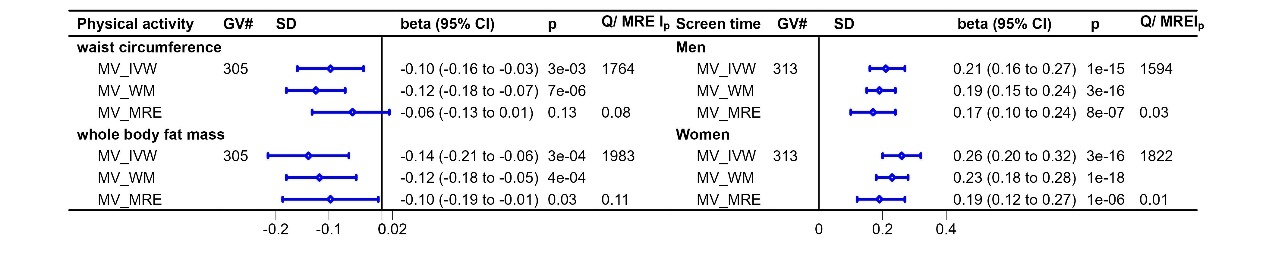
*

**Note: Causal effects of a doubling in the odds of being physically active (proxied by genetically predicted leisure-time moderate to vigorous intensity physical activity; n= 608,595) and leisure-time screen time (hours per day; n=545,263) from Wang et al. (2022) (including some participants from the UK Biobank) adjusted for education (n= 766,345) from UK Biobank on waist circumference (n= 462,166) and whole-body fat mass (n= 454,137) from the UK Biobank.**

**Abbreviations: MV: multivariable; GV#: genetic variants number; SD: standard deviation; MRE Ip: p-value for MR-Egger intercept**

**Supplementary Figure 3. Mendelian randomization estimates for the causal effects of leisure-time moderate to vigorous intensity physical activity and screen time on lifespan from the UK Biobank and LifeGen.**


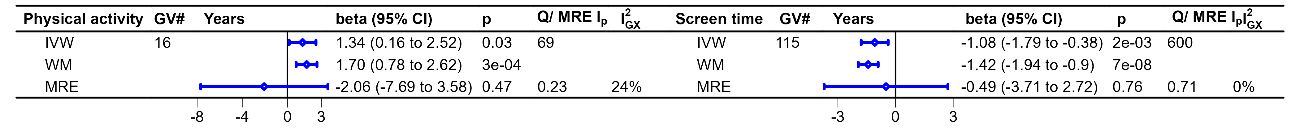


**Note: Causal effects of a doubling in the odds of being physically active (proxied by genetically predicted leisure-time moderate to vigorous intensity physical activity; n= 608,595) and leisure-time screen time (hours per day; n=545,263) from Wang et al. (2022) (including some participants from the UK Biobank) on parental lifespan (n=1,012,240) from the UK Biobank and LifeGen. Timmers’ et al. study used same way of strand as the exposure GWAS, therefore, we did not exclude palindromic SNPs.**

**Abbreviations: GV#: genetic variants number; SD: standard deviation; MRE Ip: p-value for MR-Egger intercept**

**Supplementary Figure 4. Multivariable Mendelian randomization estimates for the causal effects of leisure-time moderate to vigorous intensity physical activity and screen time adjusted for education on age at recruitment and lifespan**
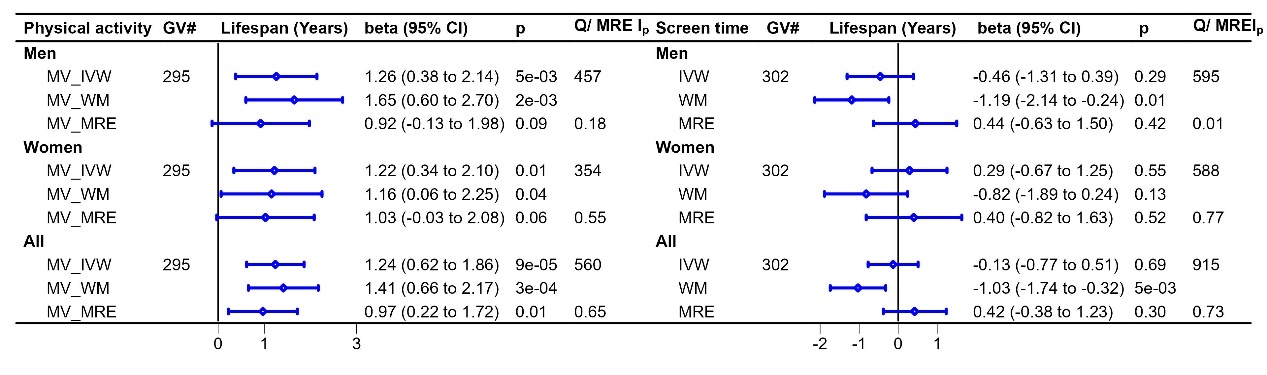


**Note: Causal effects of a doubling in the odds of being physically active (proxied by genetically predicted leisure-time moderate to vigorous intensity physical activity; n= 608,595) and leisure-time screen time (hours per day; n=545,263) from Wang et al. (2022) (including some participants from the UK Biobank) adjusted for education (n= 766,345) from the Social Science Genetic Association Consortium on age at recruitment in men (n=167,020), women (n=194,174) and overall (n=361,194) and parental attained age (fathers=415,311), (mothers=412,937) and overall (n=828,248) from the UK Biobank.**

**Abbreviations: MV: multivariable; GV#: genetic variants number; SD: standard deviation; MRE Ip: p-value for MR-Egger intercept**

**Supplementary Table 1. Detectable differences per exposure SD (80% power, 5% alpha) in lifespan and age at recruitment from univariable MR power calculations.**

| Traits | Outcome (attained age) | Estimates |
| --- | --- | --- |
| Physical activity* | Fathers’ attained age | 1.12 |
|  | Mother’s attained age | 0.85 |
|  | Parental lifespan (Combined) | 0.69 |
|  | Age at recruitment (men) | 0.57 |
|  | Age at recruitment (women) | 0.53 |
|  | Age at recruitment (overall) | 0.39 |
| Leisure screen time | Fathers’ attained age | 0.61 |
|  | Mother’s attained age | 0.46 |
|  | Parental lifespan (Combined) | 0.38 |
|  | Age at recruitment (men) | 0.31 |
|  | Age at recruitment (women) | 0.29 |
|  | Age at recruitment (overall) | 0.21 |

***The log odds of being physically active were converted to a doubling in the odds of being physically active by multiplying 0.693 (natural logarithm of two).**

**Supplementary Table 2. Bias assessment from overlapping sample using MRlap estimates of leisure-time moderate to vigorous intensity physical activity (n=608,595) and screen time (n=545,263) (hours per week) from Wang et al. (2022) (including some participants from the UK Biobank) ( on age at recruitment (men: 167,020; women: 194,174) and parental attained age (maternal n=412,937, and paternal n=415,311) from UK Biobank.**

| Exposure | Data source of exposure | Outcome | Data source of outcome | Observed effect (SE) | Corrected effect (SE) | P value for difference |
| --- | --- | --- | --- | --- | --- | --- |
| Leisure-time moderate-to-vigorous intensity physical activity | Meta-analysis of 51 studies (including UK Biobank) | age at recruitment (men) | UK Biobank (Neale lab) | 0.05 (0.05) | 0.06 (0.07) | 0.31 |
|  |  | age at recruitment (women) | UK Biobank (Neale lab) | 0.01 (0.04) | 0.02 (0.06) | 0.87 |
|  |  | age at recruitment (overall) | Metafor | 0.03 (0.03) | 0.04 (0.05) | 0.82 |
|  |  | fathers' attained age | UK Biobank | 2.06 (1.11) | 2.70 (1.59) | 0.16 |
|  |  | mothers' attained age | UK Biobank | 3.94 (1.61) | 5.38 (2.15) | 0.03 |
|  |  | Overall | Metafor | 2.66 (0.91) | 3.65 (1.28) | 0.53 |
| Leisure-time screen time | Meta-analysis of 51 studies (including UK Biobank) | age at recruitment (men) | UK Biobank (Neale lab) | 0.01 (0.03) | 0 (0.03) | 0.89 |
|  |  | age at recruitment (women) | UK Biobank (Neale lab) | -0.03 (0.02) | -0.04 (0.03) | 0.08 |
|  |  | age at recruitment (overall) | Metafor | -0.02 (0.02) | -0.02 (0.02) | 1 |
|  |  | fathers' attained age | UK Biobank | -2.74 (0.46) | -3.20 (0.69) | 1.90E-04 |
|  |  | mothers' attained age | UK Biobank | -2.59 (0.52) | -3.10 (0.78) | 9.60E-04 |
|  |  | Overall | Metafor | -2.67 (0.34) | -3.16 (0.52) | 0.43 |

**Note: Causal effects of a doubling in the odds of being physically active (proxied by genetically predicted leisure-time moderate to vigorous intensity physical activity; n= 608,595) and leisure-time screen time (hours per day; n=545,263) from Wang et al. (2022) (including some participants from the UK Biobank) on age at recruitment in men (n=167,020), women (n=194,174) and overall (n=361,194) and parental attained age for men (n=415,311), women (n=412,937) and overall (n=828,248) from the UK Biobank.**
